# The Effects of Neuromuscular Electrical Stimulation and Leap Motion–Based Exercises on Hand Function Parameters in Children With Cerebral Palsy: Study Protocol for a Randomized Controlled Trial

**DOI:** 10.2196/94705

**Published:** 2026-07-03

**Authors:** Hande Özlü Erdoğan, Gülay Aras Bayram, Sema Targıt Akbaşak, Devrim Tarakcı

**Affiliations:** 1 Department of Physiotherapy and Rehabilitation, Institute of Health Sciences, Istanbul Medipol University, Kavacık South Campus, Göztepe Neighborhood, Atatürk Avenue No: 40/16 Beykoz, İstanbul, 34815, Türkiye, +90 444 85 44; 2 Department of Physiotherapy and Rehabilitation, Faculty of Health Sciences, Istanbul Medipol University, Beykoz, Istanbul, Türkiye; 3 Department of Neurology, Central Hospital Etiler, Beşiktaş, İstanbul, Türkiye; 4 Department of Occupational Therapy, Faculty of Health Sciences, Istanbul Medipol University, Beykoz, Istanbul, Türkiye

**Keywords:** cerebral palsy, neuromuscular electrical stimulation, leap motion controller, hand function, pediatric rehabilitation

## Abstract

**Background:**

Cerebral palsy (CP) is a group of permanent but nonprogressive disorders that affect movement and posture, often accompanied by upper extremity impairments such as abnormal muscle tone, spasticity, weakness, and impaired motor control. Neuromuscular electrical stimulation (NMES) and Leap Motion–based interventions that promote neuroplasticity through different mechanisms. However, studies directly comparing these 2 approaches in children with spastic CP are limited.

**Objective:**

This study primarily aims to investigate the effects of NMES and Leap Motion–based exercise interventions on hand function in children with spastic CP and to compare the effectiveness of these 2 modalities. The secondary objective is to evaluate and compare the effects of these interventions on wrist extensor muscle activation, wrist joint range of motion, selective motor control, and hand use in daily activities.

**Methods:**

This study is a 2-arm, parallel-group randomized controlled trial with a 1:1 allocation ratio and will include 30 children aged 6‐15 years with spastic CP and distal upper extremity involvement. All participants meeting the inclusion criteria will undergo baseline assessments at T0, where wrist range of motion will be measured using an electronic goniometer; selective motor control will be evaluated using the Selective Control of the Upper Extremity Scale (SCUES); hand function will be assessed using the Jebsen-Taylor Hand Function Test; daily hand use will be evaluated using the ABILHAND-Kids questionnaire; and wrist extensor and flexor muscle activation will be assessed using surface electromyography (sEMG). All participants will then undergo a 4-week conventional exercise program (3 sessions per week, 40 minutes per session). At the end of the 4 weeks, all baseline measurements will be repeated (T1). Subsequently, participants will be randomly assigned into 2 groups: the Leap Motion group (n=15) and the NMES group (n=15). Both groups will participate in an 8-week exercise program consisting of 3 sessions per week, 60 minutes per session (including 20 minutes of either Leap Motion–based exercise or NMES application, followed by 40 minutes of conventional exercise). At the end of this 8-week program, all assessments will be repeated (T2).

**Results:**

Data collection started in January 2026. The study is expected to be completed by September 2026.

**Conclusions:**

To our knowledge, the study will be the first randomized controlled study to evaluate and compare the effects of NMES and Leap Motion–based training on hand functions in children with spastic CP. The findings are expected to contribute to evidence-based clinical practice by guiding the selection of effective upper extremity rehabilitation strategies for pediatric populations. Findings may inform future rehabilitation protocols by highlighting the benefits of integrating advanced technology-based interventions alongside conventional therapy approaches.

## Introduction

Cerebral palsy (CP) is a group of disorders arising from permanent, nonprogressive damage to the developing brain, which affects movement and postural development, leading to activity limitations. While the primary issue in CP is motor dysfunction, sensory, perceptual, cognitive, and communicative impairments may also be observed [1]. In 2000, the European Cerebral Palsy Surveillance Group conducted an epidemiological study across 16 countries to develop a more advanced classification system, classifying CP based on the distribution of motor impairment as unilateral or bilateral and according to motor type as spastic, dyskinetic, ataxic, or mixed [2]. Spastic CP results from involvement of the motor cortex and the white matter projections entering and exiting the sensorimotor area. Spastic CP is the most common motor type of CP, with a prevalence of 85%‐90%, of which one-third are unilateral and two-thirds are bilateral [3].

In children with spastic CP, upper extremity involvement is observed in approximately 50%‐70% of cases [4]. Upper extremity functionality is crucial for children’s participation in daily life and overall quality of life. While these functions play an important role in performing independence-requiring activities such as reaching, grasping, releasing, and self-care, as well as supporting the individual’s activity participation, impaired hand function negatively affects activity performance and participation skills [5]. Upper extremity disorders in CP may include abnormal muscle tone, imbalance between agonist and antagonist muscles, spasticity, alignment problems, reduced muscle strength, and impaired motor control. Consequently, hand use is typically affected, hindering the performance of daily living activities. Stereotypical postures such as wrist flexion with ulnar deviation and flexion of the fingers and thumb toward the palm make grasping and releasing skills difficult [3,6].

Conventional management of the upper extremity in children with CP typically involves physiotherapy-based interventions, including stretching, strengthening exercises, positioning strategies, splinting, casting, and movement facilitation [7]. With technological advancements in rehabilitation, evidence-based upper extremity interventions have expanded to include action observation therapy, constraint-induced movement therapy, hand-arm bimanual intensive training, repetitive transcranial magnetic stimulation, neuromuscular electrical stimulation (NMES), virtual reality (VR)–based games, and goal-directed and task-oriented training [8].

NMES has been used for various purposes, including spasticity inhibition, prevention of muscle atrophy, enhancement of muscle metabolism and enzyme activity, facilitation of isolated muscle contraction, neuromuscular facilitation of voluntary isolated muscle contraction, promotion of nerve regeneration, maintenance or improvement of normal joint movements, and modification of muscle contractile properties [9,10]. Electrical stimulation can evoke impulses that travel orthodromically through motor axons toward the target muscle, leading to muscle contraction. These impulses may also travel antidromically toward the central nervous system, potentially inducing short- and long-term neurophysiological changes in spinal reflex circuits, corticospinal pathways, and cortical networks, thereby contributing to neuroplastic modulation [11]. This study reports that NMES application to the upper extremities in children with CP is less common than its use in the lower extremity and is generally combined with orthoses or applied following botulinum toxin type A injections [10]. A systematic review reported positive effects of NMES on increasing muscle strength, reducing spasticity, improving range of motion (ROM), and enhancing hand function in children with CP [12]. However, Faccioli et al [8] indicated that there is inconclusive evidence regarding the effectiveness of NMES in improving upper extremity function in CP.

VR-based rehabilitation may support functional recovery in children with CP by promoting neuroplastic mechanisms. Repetitive, intensive, and multisensory practice can facilitate activity-dependent neural adaptation by strengthening existing neural connections and supporting the use of alternative neural pathways [13]. Through visual, auditory, and proprioceptive feedback, VR environments may engage several brain regions involved in motor planning, sensorimotor integration, attention, and movement control, thereby contributing to upper extremity motor learning [14]. The therapeutic models for upper extremity rehabilitation focus on high-intensity, repetitive, and task-specific exercises. However, combining technology and visually interactive systems with physiotherapy devices has become increasingly common, which can enhance children’s motivation and adherence to therapy [15,16]. VR interventions provide a unique, immersive environment in which patients can engage in interactive and stimulating activities tailored to their individual needs and abilities [17]. Studies in the literature that have applied VR in upper extremity rehabilitation for CP have generally focused on upper extremity dysfunction, spasticity, and muscle weakness [18,19].

Video-based game therapy models do not detect the hand and finger movements necessary for training fine motor skills. This limitation can be overcome using the Leap Motion Controller (LMC; Leap Motion, Inc), a VR method. The LMC is designed to detect, recognize, and capture hand movements and finger positions in interactive software applications. Additionally, it allows tracking of arm, wrist, and hand positions for up to 4 participants simultaneously [20]. The LMC has been reported to be highly accurate in detecting wrist flexion/extension and radial/ulnar deviation movements, but less accurate for forearm supination and pronation [21]. This study includes several studies investigating the contribution of LMC to hand function in patients with CP, stroke, spinal cord injury, and multiple sclerosis [22-25]. However, studies examining the use of the LMC in upper extremity rehabilitation for CP are quite limited.

In the literature on upper extremity interventions in CP, no study has compared LMC-based intervention with an alternative therapeutic modality. In contrast to existing studies, the present study will compare the effects of an LMC-based intervention on hand function parameters with those of NMES in children with CP, thereby evaluating the impact of 2 different interventions on hand function. This approach will enable a comparison between a traditional modality currently used in the treatment of hand function in children with CP and a recently introduced, technology-based, game-oriented therapy method. This direct comparison was considered necessary to provide clinically relevant information on which approach may be more effective for improving hand function outcomes in children with spastic CP. Furthermore, by incorporating surface electromyography (sEMG), this study aims to objectively assess changes in wrist extensor and flexor muscle activation and to explore the neuromuscular mechanisms underlying functional improvements beyond clinical outcome measures. We hypothesize that Leap Motion–based intervention will provide greater improvements in hand function parameters than NMES in children with CP.

## Methods

### Participants

The sample size was calculated using the G*Power (version 3.1.9.7; Heinrich Heine University Düsseldorf, Düsseldorf) program, with the Jebsen-Taylor Hand Function Test (JTHFT) selected as the primary outcome measure and an assumed effect size of *f*=0.5 selected with reference to a previous study [26]. The calculation was performed with a statistical power of 95% and α=.05, in line with previous research using technology-assisted intervention in CP [27], yielding a minimum required sample size of 24 participants. Considering a 20% attrition rate, a total of 30 participants, with 15 participants in each group, was considered appropriate. The sample size calculation was based only on the primary outcome measure; secondary outcomes were considered exploratory and were not independently used for power estimation.

### Inclusion Criteria for the Study Are as Follows

Diagnosis of spastic unilateral or bilateral CP confirmed by clinical and magnetic resonance imaging findings, involvement of the distal upper extremity (wrist and/or fingers), being between 6 and 15 years of age, no botulinum toxin injections or surgical interventions applied to the upper extremity within the past 6 months, Gross Motor Function Classification System (GMFCS) levels 1-3, Manual Ability Classification System (MACS) levels 1-3, upper extremity spasticity graded as 0, 1, or 1+ according to the Modified Ashworth Scale (MAS), passive wrist extension angle limitation not exceeding 10 degrees, and ability to follow instructions (mental level documented as “normal” or “mild intellectual disability” in the medical report.

Exclusion criteria were defined as follows: enrollment in a specific hand rehabilitation program at the time of this writing, presence of visual or hearing impairments, history of epilepsy, and presence of chronic, orthopedic, or other systemic conditions that may interfere with participation in the study.

### Patient Withdrawal Criteria

Participants who fail to attend 6 consecutive sessions (2 weeks), attend less than 80% of the total sessions, or develop an unexpected neurological condition (eg, epilepsy) during the study will be excluded from the analysis.

### Ethical Considerations

This study was approved by the Noninterventional Clinical Research Ethics Committee of Istanbul Medipol University (E-10840098‐202.3.02-4331, July 18, 2024). The research will be conducted in accordance with the principles of the Declaration of Helsinki. Written and verbal information will be provided to the parents of children who meet the inclusion criteria. Parents of children who agree to participate will be asked to sign an informed consent form. Children will also receive age- and developmentally appropriate information about the study procedures, and those who are unwilling to participate will not be included in the study. The informed consent form states that participation in the study is voluntary and free of charge. Participants will retain the right to withdraw from the study at any time. No additional fees will be collected from the participants, nor will any payments be made. The personal data and identity information of the participants will be kept confidential. Electronic data will be stored in password-protected files, and paper-based documents will be kept in locked cabinets accessible only to the research team. No biological specimens will be collected in this study. The data will be used solely for scientific purposes, without any commercial intent. As the intervention is considered low risk, a data monitoring committee was not established. Participant safety and data integrity will be monitored by the principal investigator.

### Study Design

**Figure 1.**
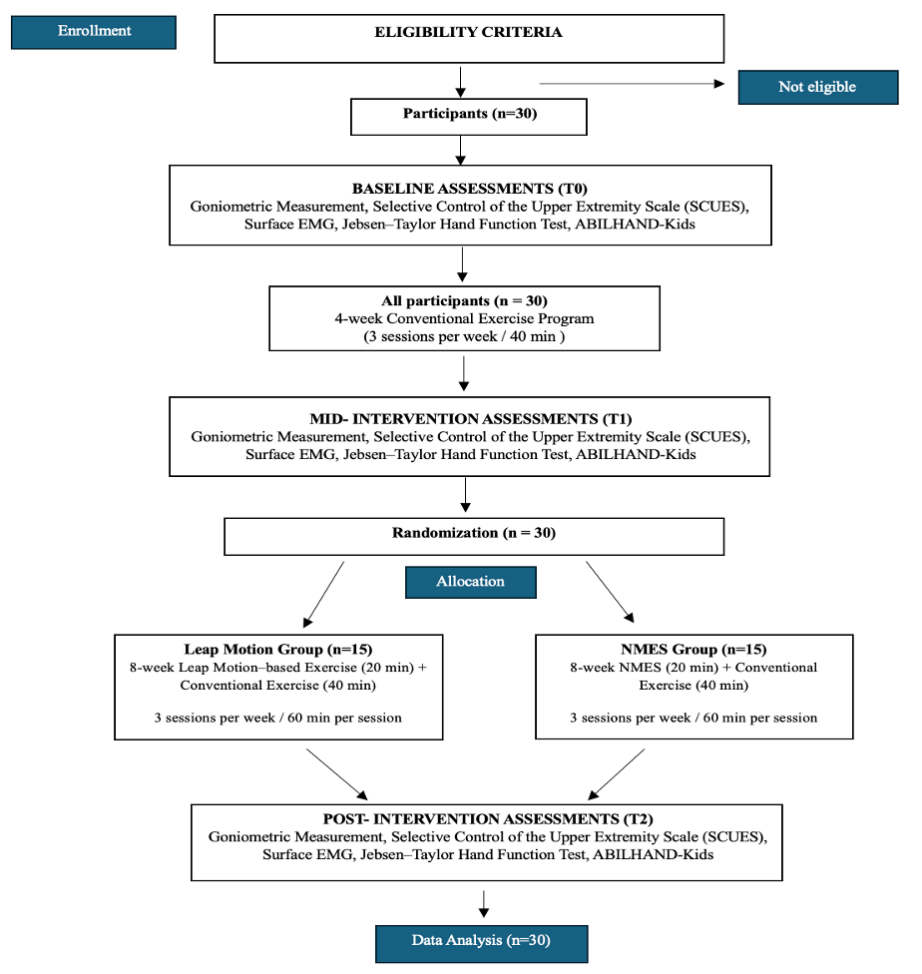
Study flow diagram. EMG: electromyography; NMES: neuromuscular electrical stimulation.

This study is registered at ClinicalTrials.gov (NCT07311018) and is designed as a 2-arm, parallel-group, randomized controlled trial (RCT) with a 1:1 allocation ratio. The study is planned to be conducted in the laboratory of the Physiotherapy and Rehabilitation Department at Istanbul Medipol University. The necessary institutional approvals have been obtained. A demographic information form will be completed for the participating children and/or their parents to collect data on age, birth history, sex, diagnosis, surgical history, medication use, family history, limb involvement, dominant side, use of assistive devices (eg, hand splint, orthosis), and whether the child is enrolled in a hand rehabilitation program at the time of this writing. The flow chart of the study is shown in Figure 1.

### Randomization and Allocation

Patients who meet the inclusion criteria and agree to participate will be randomly assigned to one of 2 groups using a computer-based randomization program [28]. Block randomization will be applied to ensure balanced group sizes, with 15 participants allocated to each group (n=15). The random allocation sequence will be generated by an independent researcher who is not involved in participant enrollment, assessment, or intervention delivery. Allocation concealment will be ensured using sequentially numbered, opaque, sealed envelopes prepared prior to the 8-week intervention. Investigators responsible for participant recruitment and outcome assessment will not have access to the randomization sequence. Due to the nature of the interventions, blinding of participants and the treating physiotherapist will not be feasible. However, outcome assessments and statistical analyses will be conducted by assessors who are blinded to group allocation.

### Eligibility Assessments

To assess eligibility according to the inclusion criteria, the following evaluations will first be performed in participating children: gross motor function will be assessed using the GMFCS, manual ability level will be evaluated using the MACS, and upper extremity muscle spasticity will be measured using the MAS.

#### GMFCS

The GMFCS is based on the self-initiated movements of children with CP, emphasizing sitting, transfers, and mobility. This classification system consists of 5 levels. The distinctions between levels are determined primarily by functional limitations, the need for assistive mobility devices (such as walkers, crutches, wheelchairs, or canes), and, to a lesser extent, by the quality of movement. During the assessment, gross motor skills are evaluated considering both the child’s abilities and the limitations in motor function. Children at Level 1 walk without restrictions, those at Level 2 walk with limitations, children at Level 3 walk using handheld mobility devices, those at Level 4 have limited self-mobility and may use powered mobility, and children at Level 5 are transported in a manual wheelchair. Considering the age-related differences in motor function, the GMFCS is categorized for children less than 2 years, 2-4 years, 4‐6 years, 6‐12 years, and 12‐18 years [29].

#### MACS

MACS was developed to classify how children with CP use their hands when handling objects in daily activities, reflecting typical hand performance rather than maximal capacity. The classification describes how children usually use their hands to handle objects at home, at school, and in the community, rather than their best possible performance. It evaluates the ability to handle objects in children with CP aged 4‐18 years. The system consists of 5 levels: children at Level 1 can handle objects easily and successfully, although they may have difficulties with speed and accuracy in activities. Children at Level 5, however, cannot handle objects effectively and have severely limited abilities, requiring support to perform even the simplest hand activities [30].

#### MAS

The severity of spasticity will be evaluated using the MAS**,** which is widely used to quantify resistance perceived during passive movement [31]. The MAS grades muscle tone on a 0‐4 scale. Starting from 0: no increase in muscle tone; 1: slight increase in muscle tone, characterized by a minimal resistance at the end of the ROM; 1+: slight increase in muscle tone, characterized by a catch followed by minimal resistance through less than half of the ROM; 2: more marked increase in muscle tone through most of the ROM, but the affected part is easily moved; 3: considerable increase in muscle tone, making passive movement difficult; and 4: affected part rigid in flexion or extension. In children with CP, spasticity most frequently involves the upper extremity muscles, particularly the shoulder flexors, adductors, and internal rotators; elbow flexors; forearm pronators; wrist flexors; and finger flexors [32,33]. Accordingly, spasticity of these muscle groups in both upper extremities will be assessed using the MAS in this research.

### Outcome Measures

The primary outcome of this study is hand function, and the secondary outcomes are wrist extensor and flexor muscle activation, wrist ROM, wrist selective motor control, and hand use in daily activities. The baseline assessments (T0) will be done after enrollment. Before randomization, all participants will complete a 4-week standardized upper extremity–focused conventional therapy program, which will be conducted as a baseline standardization period to reduce the potential influence of previous routine rehabilitation programs. This phase will not be considered part of the randomized intervention comparison. Postconventional exercise assessments (T1) will be conducted after a 4-week conventional therapy program. Randomization will be performed after the T1 assessment to allow comparison of the additional effects of NMES and Leap Motion–based training when added to the standardized conventional exercise program. The final assessments (T2) will be performed after 8-week NMES and Leap Motion–based interventions. The main comparison will be based on the change from T1 to T2 between the intervention groups. A randomized controlled design was selected to minimize selection bias and to ensure balanced baseline characteristics between the intervention groups. The inclusion of 3 assessment time points (T0, T1, and T2) allows differentiation between the effects of conventional therapy and intervention-specific effects.

### JTHFT

The JTHFT is a timed, 7-subtest assessment designed to evaluate hand functionality. The test is derived from common daily activities and includes the following tasks: writing a standardized 24-letter sentence, turning 5 cards, picking up small objects, picking up 5 beans using a spoon, stacking 4 checkers on a test board, moving 5 large empty cans, and moving 5 full cans. Since the study population includes children who may not yet be literate according to the age criteria, the first subtest (writing a sentence) will be omitted from the evaluation. Each subtest allows a maximum of 120 seconds for successful completion. All subtests are first performed with the nondominant hand, followed by the dominant hand. The validity and reliability of the JTHFT in children with CP aged 6‐18 years have been established by Tofani et al [34].

### Selective Control of the Upper Extremity Scale

The Selective Control of the Upper Extremity Scale (SCUES) was developed to assess selective voluntary motor control (SVMC) of the upper extremity and represents the first scale specifically designed for this purpose. It is a practical and user-friendly, video-based assessment tool that can be administered in less than 15 minutes. During the evaluation, the physiotherapist first demonstrates the required joint motion passively within the desired ROM and then asks the child to perform the movement 3 times at each joint. The test provides the opportunity to assess selective movements in all upper extremity joints, including the shoulder, elbow, forearm, wrist, and fingers. Each of the 5 joint regions is scored on a 4-point ordinal scale: absence of SVMC (0 points), moderately reduced SVMC (1 point), mildly reduced SVMC (2 points), and normal SVMC (3 points). The total SCUES score ranges from 0 to 15, with higher scores indicating better SVMC [35]. In the present study, only the selective control of the upper extremities will be assessed bilaterally.

### sEMG

**Figure 2.**
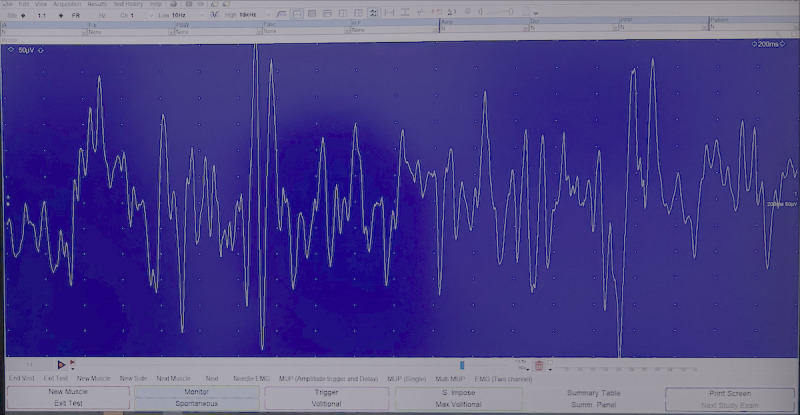
Screenshot of the surface electromyography (sEMG) recording interface.

**Figure 3.**
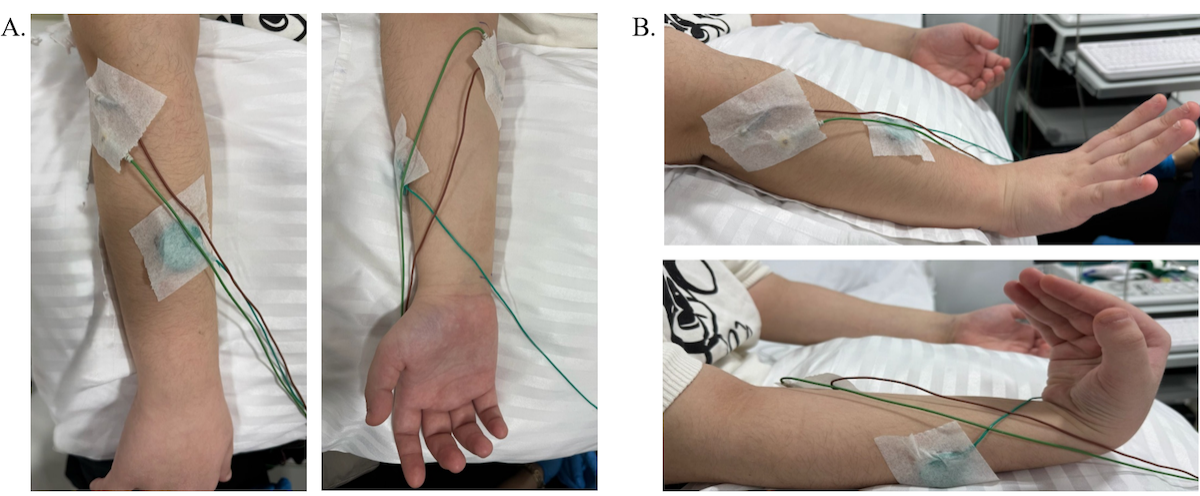
Surface electromyography (sEMG): (A) electrode placement over the wrist extensor and wrist flexor muscle groups; (B) testing procedure.

sEMG is based on the principle that there is a linear relationship between sEMG signal amplitude and muscle contraction force, with signal amplitude increasing as muscle force increases [36]. sEMG can be used as an outcome measure in children with CP, providing quantitative information related to motor activation when the processes are correctly analyzed [37]. In the present study, muscle activation will be assessed using the Natus UltraPro S100 (Electromyography (EMG)/Nerve Conduction Studies (NCS)/Evoked Potentials (EP) neurodiagnostic system; Natus Medical Incorporated). Surface electrodes will be applied to measure muscle activity. The child will be asked to perform maximum voluntary contraction, and the sEMG signals from the muscles will be recorded for 10‐15 seconds, followed by a 10-second rest period (Figure 2) [38]. Electrodes will be positioned parallel to the muscle fibers over the wrist extensor and flexor muscle bellies Figure 3A using anatomical landmarks in accordance with the guidelines of the Surface Electromyography for the Noninvasive Assessment of Muscles (SENIAM) [39]. To ensure consistency across assessment time points, electrode placement will be performed by the same neurology specialist.

The sEMG signal processing procedure will be performed as follows: raw sEMG signals will be processed using a 20 Hz infinite impulse response Butterworth high-pass filter and then a 500 Hz infinite impulse response Butterworth low-pass filter will be applied to reduce movement artifacts and high-frequency noise. Subsequently, root mean square values will be derived from the filtered sEMG signals using consecutive 0.1-second time windows for signal analysis. The filtered sEMG signals will then be analyzed using root mean square amplitude values expressed in microvolts (µV), without additional normalization to maximum voluntary contraction. Each measurement will be repeated 3 times, and activation of the wrist extensor and flexor muscles will be recorded (Figure 3B). To minimize signal variability in the pediatric population, standardized verbal instructions will be provided, measurements will be repeated with rest intervals, recordings with visible movement artifacts will be excluded, and the mean value of valid trials will be used for analysis.

### Goniometric Assessment

Active wrist ROM will be assessed with Joints Digital Goniometer 360 ^0^ (JTECH Medical) [40]. In addition to digital goniometer measurements, wrist ROM will be assessed with a Becure HandROM (Becure GmbH) application integrated into the LMC. Wrist flexion, extension, ulnar deviation, and radial deviation ROM will be measured bilaterally. Each measurement will be repeated 3 times, and the mean value will be used for statistical analysis.

### ABILHAND-Kids

The ABILHAND-Kids questionnaire will be used to assess hand use in daily life activities. ABILHAND-Kids is designed to measure upper extremity function in children with CP aged 6‐15 years and to evaluate unimanual and bimanual activities of the upper limbs as comprehensively as possible [41]. The questionnaire consists of 21 items covering both unimanual and bimanual tasks and will be completed by parents using a 3-point scale: 0-cannot perform the activity, 1-performs the activity with difficulty, and 2-performs the activity easily. The 21 items include daily tasks such as fastening pants buttons, fastening shirt buttons, opening a jar, zipping a jacket, sharpening a pencil, putting on a backpack, zipping pants, rolling up a sweater sleeve, applying toothpaste to a toothbrush, opening a bottle cap, opening a chip bag, opening a toothpaste tube, washing the upper body, filling a glass with water, opening a bread box, taking off a T-shirt, wearing a hat, retrieving coins from a pocket, opening a chocolate wrapper, turning on a bedside lamp, and opening jacket snaps. ABILHAND-Kids is a test specifically developed for children with CP and requires no preparatory phase. The Turkish validity and reliability study in children with CP was conducted by Sahin et al [42].

### Interventions

This study consists of 5 sequential phases. In the first phase, eligibility of the participants will be assessed according to the predefined inclusion criteria and written informed consent will be obtained. In the second phase, baseline assessments will be conducted (T0). In the third phase, all participants will undergo a 4-week conventional exercise program before randomization, followed by midintervention assessments (T1). In the fourth phase, participants will be randomized into either the Leap Motion–based exercise group or the NMES group, and both groups will receive these interventions in addition to conventional exercise for 8 weeks. In the fifth and final phase, all outcome measures will be reassessed after completion of the intervention period, and the study will be closed out (T2).

Treatment adherence will be monitored using standardized follow-up forms, including session attendance, completed sessions, missed sessions, and adherence to the intervention protocol. All interventions in the NMES and Leap Motion–based training groups as well as the conventional exercise program will be conducted by the same pediatric physiotherapist with at least 8 years of experience. In addition, external rehabilitation programs will be discontinued after enrollment in this study.

### Conventional Exercise Program

All participants who meet the inclusion criteria and provide informed consent will be enrolled in a 4-week conventional exercise program. The conventional exercise program will be administered 3 times per week, with each session lasting 40 minutes. The program will be structured at the beginning of the treatment period by selecting exercises from a predefined pool of neurodevelopmental treatment–based upper extremity exercises, including bilateral and unilateral upper extremity weight-bearing activities for tone regulation, passive stretching, muscle strengthening, and active ROM exercises. To improve fine motor skills, activities targeting reaching, grasping, releasing, carrying, in-hand manipulation skills, and bimanual use will also be included. The exercises will be selected and incorporated into the program based on each child’s level of impairment and clinical needs. After randomization, the selected exercises will be applied consistently across sessions for 8 weeks in addition to the group-specific interventions. While the session duration and frequency will remain standardized, exercise intensity will be adjusted by modifying the number of repetitions according to the child’s tolerance and performance.

### NMES Group

**Figure 4.**
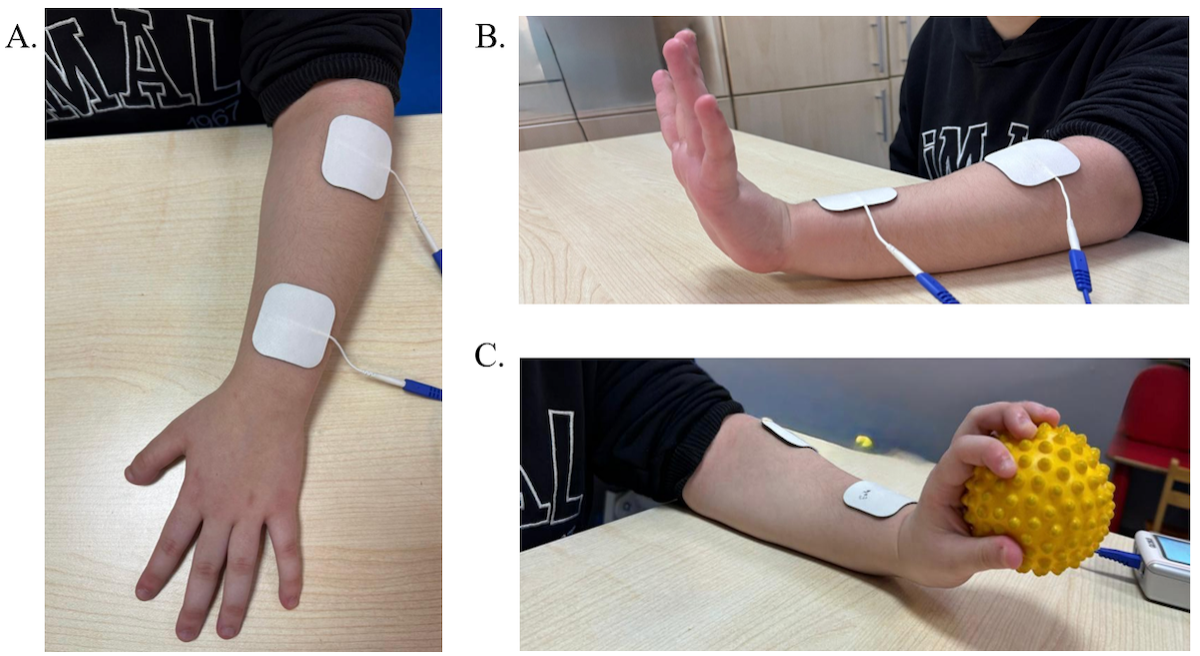
Neuromuscular electrical stimulation (NMES) applications: (A) passive; (B) with active wrist extension; and (C) with functional task.

For NMES application, a portable 4-channel device (Globus Premium-400; Globus Corporation Srl) will be used. The NMES provides a biphasic waveform, with a pulse duration ranging between 50 and 300 μs and a frequency range of 1‐120 Hz. In spastic CP, stimulation frequencies between 10 and 60 Hz are generally recommended to elicit motor activation, although frequencies above 50 Hz may induce muscle fatigue [43]. In this study, stimulation parameters will be set to a pulse duration of 300 μs, a frequency of 30 Hz, and an on/off ratio of 10 s/10 s [44]. The stimulation intensity will be increased until visible muscle contraction is observed and will then be adjusted to the maximum level tolerated by the participant. The intervention will be administered for 20 minutes per session, in addition to the conventional exercise program, over a period of 8 weeks. Electrode placement will be as follows: the inactive electrode on the dorsal aspect of the wrist, and the active electrode on the lateral forearm over the bellies of wrist extensor muscles (extensor carpi radialis longus and brevis, extensor carpi ulnaris). For participants with bilateral spastic CP, stimulation will be applied to the dominant side, whereas for those with unilateral spastic CP, it will be applied to the affected side. During the first 2 weeks, NMES will be applied passively (Figure 4A). In subsequent sessions, active participation will be encouraged by asking participants to perform wrist extension synchronously with the stimulation (Figures 4B and 4C). During the sessions, children will be monitored for pain, discomfort, muscle fatigue, skin irritation, redness at the electrode site, or any adverse response. Any adverse events will be recorded, and the session will be stopped if necessary.

### NMES Training Protocol

In this group, each session will include 20 minutes of NMES and 40 minutes of conventional exercises. The NMES intervention will be applied progressively over an 8-week period as follows:

- Weeks 1‐2: Participants will receive passive NMES application.
- Weeks 3‐5: NMES will be combined with active wrist extension. During the rest phase, the wrist will be maintained in a neutral position, while in the stimulation phase, participants will be instructed to actively perform wrist extension movements.
- Weeks 6‐8: NMES will be integrated into functional tasks. During the rest phase, participants will practice grasping objects of varying sizes (eg, balls), while in the stimulation phase, they will be instructed to release the object through active wrist extension.

### Leap Motion–Based Exercise Group

The Leap Motion–based VR system consists of a personal computer and an LMC, which are connected via a USB. Using infrared technology, the Leap Motion device allows for the tracking of multiple hand and finger movements without the need for markers or data gloves [20]. The Fizyosoft Becure company has developed software integrated into the Leap Motion system, creating task-oriented games designed to improve grasping ability and hand activities in individuals with upper extremity limitations. These games represent the first Turkish-language software developed for the Leap Motion system [45]. Each session of the VR intervention will last for 20 minutes and will use games developed within the Becure HandROM platform.

#### Leap Ball

Involves grasping a ball by activating all interphalangeal flexors followed by interphalangeal and wrist extensors and then placing the ball into designated holes.

#### bRabbit

Requires repetitive wrist flexion and extension to jump over snakes and hide from flying birds.

#### Pong

Focuses on repetitive wrist flexion and extension to hit rapidly incoming balls, with increasing speed and frequency.

**Figure 5.**
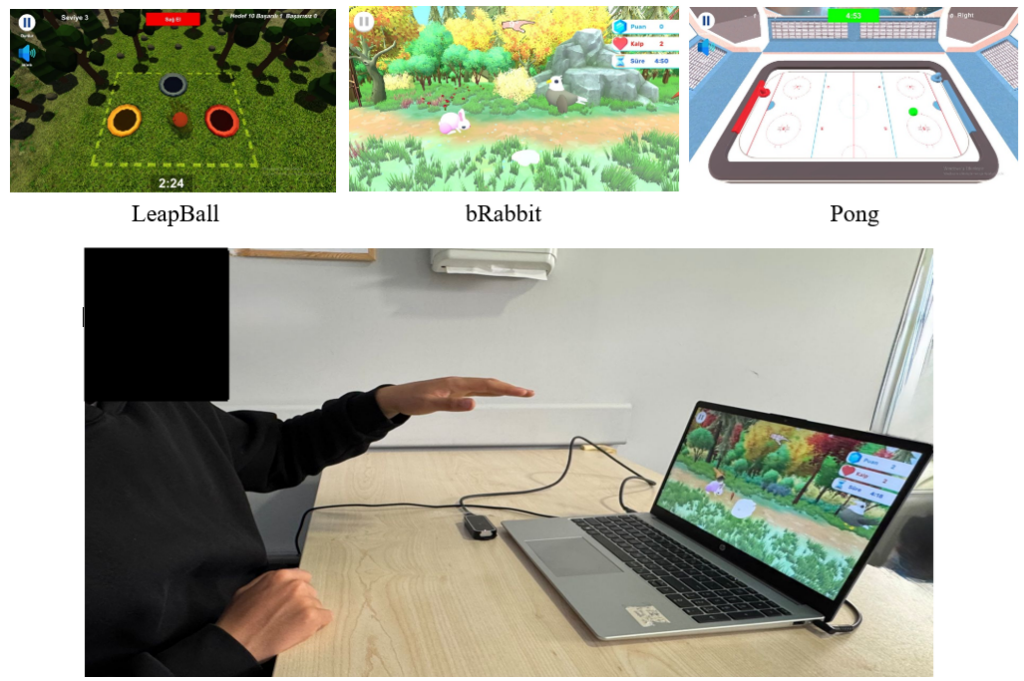
Leap motion-based serious game screenshots and game-play.

All games will be progressively adapted to ensure advancement, with modifications specific to each game. The Pong game is expected to be more challenging compared to the others; therefore, it will be introduced starting from the third week of intervention. Prior to the intervention, children will be introduced to the Leap Motion sensor and games, including instructions about optimal positioning relative to the sensor and game-play mechanics. They will also be provided an opportunity to practice before the actual intervention begins. During the sessions, compensatory movements will be prevented by the supervising physiotherapist. Each game will be adjusted to the child’s needs, starting from an appropriate difficulty level. Progression across weeks will be achieved by modifying parameters (such as game speed, trial repetitions, and the size or number of balls/holes) (Figure 5). During sessions, children will be monitored for fatigue, discomfort, dizziness, or any adverse response. Rest breaks will be provided when needed. Any adverse events will be recorded, and the session will be stopped if necessary.

### Leap Motion–Based Training Protocol

In this group, each session will consist of 20 minutes of Leap Motion–based training and 40 minutes of conventional exercises. The Leap Motion–based intervention will be conducted over an 8-week period with progressive difficulty as follows:

- Weeks 1‐2: participants will begin with introductory sessions consisting of VR-based exercises using the Leap Ball and bRabbit games.
- Weeks 3‐5: training difficulty will be increased by adjusting game parameters such as speed, number of repetitions, number, and size of balls/targets, according to each child’s individual level. The game set will include Leap Ball, bRabbit, and Pong games.
- Weeks 6‐8: the same progression strategy will be maintained, with further increases in task complexity through modifications in game parameters (eg, speed, repetitions, number, and size of balls/targets). The game set will again include Leap Ball, bRabbit, and Pong games.

### Statistics

Statistical analyses will be performed using SPSS for Windows (version 25.0; IBM Corp). Descriptive statistics will be calculated for all variables. Continuous variables with normal distribution will be presented as mean (SD), continuous variables without normal distribution as median (minimum-maximum), and categorical variables as frequency and percentage. The normality of continuous variables will be assessed using the Shapiro-Wilk test. The primary intervention effects will be analyzed using a 2-way repeated measures ANOVA to evaluate the main effects of group and time, as well as the group×time interaction. When significant main or interaction effects are detected, post hoc pairwise comparisons will be performed with Bonferroni correction for multiple comparisons. For variables that do not meet the assumptions for parametric analysis, appropriate nonparametric alternatives will be used. Categorical variables will be analyzed using the chi-square test or Fisher exact test, depending on the expected cell counts. The primary analysis will be conducted as a complete-case analysis, including only participants who complete all assessment time points. Dropouts and missing data will be documented. Effect sizes will be reported to support the interpretation of the magnitude of intervention effects. Partial eta squared will be reported for repeated measures ANOVA results. A *P* value of <.05 will be considered statistically significant.

## Results

The study was approved by the institutional ethics committee on July 18, 2024. It was subsequently funded by the Istanbul Medipol University Scientific Research Project (project number 2026‐08) in January 2026, after which participant recruitment commenced. As of June 2026, 11 participants had been enrolled. Data analysis will be conducted once all participants have completed the study protocol. The study is expected to be completed by September 2026.

## Discussion

This study is expected to provide clinically relevant information on the comparative effects of NMES and Leap Motion–based training on hand function parameters in children with spastic CP. In this context, changes in hand function, wrist extensor and flexor muscle activation, wrist ROM, wrist selective motor control, and hand use in daily activities will be evaluated. Although both interventions are expected to improve hand function parameters, Leap Motion–based training is anticipated to show greater improvements than NMES.

Several studies have demonstrated the potential benefits of NMES in upper extremity rehabilitation in children with CP [44,46-48]. Yıldızgören et al [47] reported that adding NMES to conventional therapy effectively improved active wrist extension ROM, spasticity, and hand function in children with CP. Açıkbaş et al [44] demonstrated significant improvements in shoulder and wrist joint ROM following NMES applied to the wrist extensor muscles in children with CP. However, findings related to spasticity reduction and functional transfer to daily activities have been inconsistent across studies.

Kamper et al [9] reported improvements in wrist extension ROM and muscle strength following NMES applied to the wrist flexor and wrist extensor muscles, although changes in spasticity and passive resistance were less pronounced. In a pilot study evaluating NMES applied to the wrist extensor muscles following botulinum toxin injections and prior physiotherapy and occupational therapy, improvements in hand function, spasticity, and ROM were observed; however, the differences were not statistically significant [49].

Some studies highlight the applicability of NMES as part of a multimodal rehabilitation approach, underlining its potential to enhance functional outcomes when combined with other therapeutic interventions [46,48]. In a study investigating the use of NMES and dynamic splints for upper extremity spasticity management in children with CP, improvements in functional outcomes were reported when both modalities were applied [48]. Xu et al [46] reported that the combined application of constraint-induced movement therapy and NMES to the extensor carpi radialis and extensor digitorum muscles resulted in significant improvements in upper extremity functional test results. In the literature, NMES protocols vary considerably in terms of stimulation parameters, target muscles, and treatment duration, which limits direct comparisons and may contribute to heterogeneity in reported outcomes.

Studies examining the use of the LMC in upper extremity rehabilitation for children with CP are limited. Tarakcı et al [45] evaluated upper extremity functions and demonstrated that LMC-based exercises may serve as an effective alternative treatment option for improving functional outcomes in physically disabled children and adolescents. Research comparing neurodevelopmental therapy with video-based game therapy (using the Nintendo Wii [Nintendo Co, Ltd] and LMC) reported superior effects on hand skills in the video-based therapy group, while both interventions demonstrated beneficial effects on grip strength and functional abilities [22]. Daliri et al [27] reported that comparing LMC-based rehabilitation with conventional rehabilitation methods found greater improvements in grip, lateral, and palmar pinch strengths in the LMC group.

In the literature on upper extremity interventions in CP, no study has compared LMC intervention with another method. Unlike existing studies, this research will compare the effects of LMC intervention on hand function parameters with a group of children receiving NMES, thereby evaluating the impact of 2 different interventions on hand function. This will enable the comparison of a traditional modality currently used in the treatment of hand function in children with CP with a game-based therapy method that has recently emerged with technological advancements. In addition, although NMES has been applied predominantly in a passive manner in previous RCTs, the present protocol proposes an NMES approach consisting of an initial passive stimulation period followed by active wrist extension exercises. This strategy was designed to incorporate motor learning principles into the NMES intervention and to provide a more methodologically balanced comparison with the inherently active, sensorimotor nature of the Leap Motion–based exercise intervention.

Selective motor control ability of the upper extremity is considered a fundamental factor for performing many activities such as eating, participating in daily life activities, engaging in self-care tasks, and writing [50,51]. Therefore, assessing selective motor control of the wrist is of great importance when evaluating hand functions. In this research, the effects of NMES and LMC interventions on upper extremity selective motor control in children with CP will also be examined, representing another unique aspect of the study. By incorporating the assessment of selective wrist motor control, the present protocol aims to provide a more comprehensive evaluation of upper extremity function.

Weakness of prolonged wrist extensors combined with increased flexor spasticity has been associated with poorer hand function, and interventions aimed at facilitating wrist extension to modify muscle characteristics related to hand dysfunction have been suggested to improve hand function in children with CP [52]. In studies, RCTs involving LMC interventions in CP have generally assessed muscle strength using a hand dynamometer and a pinch meter to measure grip strength [22,27,45]. In RCTs involving NMES interventions, grip strength has been evaluated using a hand dynamometer [48], computerized dynamometer [53], and sphygmomanometer [46]. However, only one randomized controlled study has assessed wrist extensor muscle activation using sEMG [38]. This highlights that the use of objective, muscle-specific activation measures in CP has not been widely implemented in RCTs. The present research aims to evaluate isolated wrist extensor and flexor muscle activation using sEMG and demonstrate the effects of both interventions on the wrist extensor muscles objectively.

For clinical interpretation, changes in JTHFT scores will be considered in relation to previously reported minimal clinically important difference values in children with CP, including 54.7 seconds for the more affected hand and 20.9 seconds for the less affected hand [26]. For secondary outcomes, established minimal clinically important difference values are limited or not clearly defined in this population; therefore, the magnitude and direction of change, effect sizes, and available measurement properties will be considered when interpreting clinical relevance.

Several potential limitations of this study should be acknowledged. First, the relatively small sample size should be considered when interpreting the findings. Due to the nature of the interventions, blinding of participants and therapists is not feasible, which may introduce performance bias. Because all participants will receive the same standardized conventional therapy before randomization, this phase is expected to reduce between-participant variability rather than introduce differential bias between groups. However, the possible influence of this pre-randomization phase on subsequent responses to the randomized interventions will be considered when interpreting the findings. The absence of long-term follow-up assessments restricts the ability to determine the persistence of intervention effects over time. Additionally, the game-based and interactive nature of the Leap Motion–based intervention may increase participant motivation and engagement, which may introduce potential motivational bias. Future studies with larger sample sizes, longer follow-up periods, and combined or additive intervention models may further clarify the long-term and comparative effects of these approaches.

After completion of the study, the findings will be disseminated through peer-reviewed publication and scientific meetings, with the aim of informing evidence-based upper extremity rehabilitation practices for children with CP.

In conclusion, this study is expected to contribute to the literature by providing clinically relevant evidence on the comparative effects of 2 interventions based on different neuroplasticity stimulation mechanisms in pediatric upper extremity rehabilitation. The integration of clinical outcome measures with objective sEMG assessment may offer a more comprehensive understanding of wrist muscle activation and functional changes in children with spastic CP. The findings may guide the selection of intervention methods, support evidence-based rehabilitation protocols, and contribute to the applicability of different modalities in clinical practice.

## Supplementary material

10.2196/94705 Checklist 1 CONSORT-EHEALTH (V 1.6.1) checklist.

10.2196/94705 Checklist 2 SPIRIT checklist.

## Abbreviations

CONSORT-EHEALTH: Consolidated Standards of Reporting Trials of Electronic and Mobile Health Applications and Online Telehealth
CP: cerebral palsy
GMFCS: Gross Motor Function Classification System
JTHFT: Jebsen-Taylor Hand Function Test
MACS: Manual Ability Classification System
MAS: Modified Ashworth Scale
NMES: Neuromuscular Electrical Stimulation
RCT: randomized controlled trial
ROM: range of motion
SCUES: Selective Control of the Upper Extremity Scale
sEMG: Surface Electromyography
SENIAM: Surface Electromyography for the Noninvasive Assessment of Muscles
SPIRIT: Standard Protocol Items: Recommendations for Interventional Trials
SVMC: selective voluntary motor control
VR: virtual reality
